# Relationship between internet addiction and sleep disturbance in high school students: a cross-sectional study

**DOI:** 10.1186/s12887-020-02275-7

**Published:** 2020-08-11

**Authors:** Mikiko Tokiya, Osamu Itani, Yuichiro Otsuka, Yoshitaka Kaneita

**Affiliations:** 1 Department of Public Health and Epidemiology, Faculty of Medicine, Oita University, 1-1 Idaigaoka, Hasama-machi, Yufu-shi, Oita 879-5593 Japan; 2 Division of Public Health, Department of Social Medicine, Nihon University School of Medicine, 30-1 Ohyaguchikami-machi, Itabashi-ku, Tokyo, 173-8610 Japan

**Keywords:** Internet addiction, Sleep disturbance, Youth, Pittsburgh sleep quality index, Young diagnostic questionnaire

## Abstract

**Background:**

The increase in the number of Internet users has increased Internet dependence worldwide. In adolescents, this dependence may interfere with sleep, which is important for the development of psychophysiological capabilities. However, few large-scale surveys have described the relationship between Internet addiction (IA) and sleep disturbance using standardized questionnaires. We conducted a survey in one prefecture in Japan to determine the relationship between sleep disturbance and IA in adolescents based on the categories of the Young Diagnostic Questionnaire (YDQ).

**Methods:**

In 2016, high school students (*N* = 10,405, age range: 15–16 years) in all 54 daytime high schools in the selected prefecture were surveyed using a self-administered questionnaire. Participants with scores > 5.5 points on the Japanese version of the Pittsburgh Sleep Quality Index were defined as having a sleep disturbance. IA was evaluated using the YDQ: Participants with five to eight YDQ items present were classified as having IA; those with three or four items present were classified as “at risk of IA”; and those with two or less YDQ items were classified as “non-IA”. Multiple logistic regression analysis was performed with sleep disturbance as the dependent variable, IA as the explanatory variable, and adjustments for eight other variables.

**Results:**

High YDQ scores were associated with a high prevalence of sleep disturbance in boys and girls. These findings persisted after controlling for other factors in the multiple regression model.

**Conclusions:**

Among Japanese adolescents, there was a significant independent relationship between IA and sleep disturbance.

## Background

The Internet is a network that connects information devices across the world to provide convenient information and communication technology that enables various activities, ranging from exchanging electronic mail and information to shopping. In 2016, when this study was conducted, 48% of all people worldwide used the Internet [1]. In this context, a large survey of Japanese youth found that 6.2% of boys and 9.8% of girls presented problematic Internet use [2].

Internet addiction (IA) has been defined as “an impulse-control disorder that does not involve an intoxicant” [3]. This survey examined the concept of IA. Generalized IA is a concept that was initially introduced by Davis et al. [4] based on a cognitive-behavioral model. More recently, a meta-analysis of 89,281 individuals in 31 countries from 1996 to 2012 reported an IA prevalence of 6%, with a median age of 18.42 years (standard deviation [SD], 5.02; range, 12–41) [5]; individuals aged between 15 and 24 years account for approximately 25% of Internet users worldwide [1]. Moreover, this age range includes adolescents, which means that policies regarding IA must consider this population.

A previous study on IA among adolescents reported a significant relationship between this addiction and psychiatric disturbances, including “interpersonal sensitivity,” “depression,” “anxiety,” “hostility,” and “psychoticism.” [6]. Furthermore, adolescent IA has been reported to be a risk factor for problematic alcohol use in adulthood [7, 8]. Recent studies using functional magnetic resonance imaging have reported that IA is related to structural and functional damage in the prefrontal cortex [9]. With such severe negative impacts on life, the seriousness of this problem has been increasingly recognized, and several epidemiological studies have been conducted to determine factors related to IA. For example, a study that examined the data of 100,000 Japanese youth found that IA was related to the frequency and amount of alcohol consumption [10]. A study of 2620 Chinese high school students reported a relationship between IA and emotional anxiety and a lack of empathy [11]. Excessive smartphone use may be associated with musculoskeletal discomfort and mental health problems [12, 13].

For adolescents, sleep behavior is a component of daily life that has a major impact on physical and mental health [14]. Moreover, adolescent sleep is important because of its significant effects on the development of vital psychophysiological functions, including behavior, emotions, and attention [15–21]. Therefore, it is important to investigate the relationship between sleep and IA. Some studies have reported an association between IA and depression and sleep disturbance [22, 23], nighttime sleep duration and subjective insomnia [24], poor sleeping habits [25], smartphone dependence [26], and sleep quality [27]. Sleeping habits are associated with other lifestyle habits, such as extracurricular activities and skipped meals [28, 29]. However, the relationship between IA and sleep disturbance in adolescents has not yet been comprehensively investigated, because few large-scale surveys have been undertaken using standard indicators, such as the Pittsburgh Sleep Quality Index (PSQI) [30].

We hypothesized that sleep disorders in puberty are associated with a general degree of Internet dependence and that this association is also attributable, in part, to other lifestyle habits. It is important to take lifestyle habits into account, because they can weaken the relationship between sleep disorders and Internet dependence. Therefore, we conducted an epidemiological study to determine the relationship between IA and sleep disturbance in Japanese high school students.

## Methods

### Study population and design

After obtaining the consent of the President of the Association of High School Principals and the prefectural Education Bureau of one prefecture in Japan, we sent requests for participation to the principals of all 54 daytime high schools within the prefecture and sent the following documents via the postal service to each principal: (1) letter requesting cooperation; (2) planning document containing the study purpose and method; and (3) the questionnaire to be used in the study. We specified that a self-administered questionnaire form would be used in the survey, with assured protection of respondent privacy. A total of 10,405 students were registered at the 54 daytime high schools.

The survey procedure was as follows: (1) the teachers distributed the following three items: an explanatory document, a self-administered questionnaire, and an envelope; (2) after filling in their responses in the questionnaire form, the surveyed students placed the completed questionnaire form in the provided collection envelope and sealed the envelope; (3) the teachers collected the sealed envelopes; and (4) the envelopes containing the self-administered questionnaires were not unsealed and opened until they were used for data entry at the research facility. The survey period was from June to December 2016.

### Measurements

The questionnaire collected information on participant demographic characteristics, sleep disturbance, and IA.

#### Demographic characteristics

Data were collected on the name of the school, grade, and name and gender of the student. After recording the school names, participants were classified according to whether they were attending a public school or a private school. Questions on daily-life habits included school-commute time, time spent engaging in school sports or clubs, time spent on study outside school hours, television-viewing time, and skipped meals. These questions were similar to those used in previous studies among adolescents [10, 31–33] (Additional file 1). The items on emotions and perceptions were measured by assessing depressed mood and school-life satisfaction.

We adopted the measure of depressed mood used in previous studies [31, 33]. The question was: “Over the past 30 days, did you have feelings of heaviness or depression more than usual?” We measured school-life satisfaction using a 2013 survey conducted by the Cabinet Office on the attitudes of young people in Japan and other countries [34]. The question was: “Are you satisfied or dissatisfied with your school life?”

#### Measurement of sleep disturbance: Japanese version of the Pittsburgh sleep inventory

Sleep disturbance was evaluated using the Japanese version of the PSQI (J-PSQI) [35–37]. Based on previous studies, scores ≥5.5 points on the J-PSQI were considered indicative of sleep disturbance [35–37].

#### Measurement of IA: Japanese version of the Young diagnostic questionnaire

We measured IA using the Young Diagnostic Questionnaire (YDQ) [3, 38–44]. We used the Japanese version of the YDQ (J-YDQ) which has been used in previous studies [31]. The J-YDQ is an evaluation tool composed of eight questions, which are scored as 1 point for “yes” and 0 points for “no,” with the total score ranging from 0 to 8 points. The participants were grouped into three categories: “IA,” if they scored 5–8 points, “at-risk,” if they scored 3–4 points, and “no IA,” if they scored 0–2 points [25, 33, 39, 43, 45, 46].

### Ethical considerations

The participation of students in the present study was voluntary. As our cohort included 15- to 16-year-old adolescents, we obtained written informed consent directly from the students or their parents when their supervising teacher confirmed that their judgment was acceptable or he/she thought that the parents’ consent was necessary, respectively. The following statements were included in the consent document distributed to students and their families: (1) the survey was part of an epidemiological study and involved neither an evaluation for school grading nor any type of punishment; (2) students were free to cooperate in the survey, and failure to cooperate would not incur any disadvantage; (3) the school teachers would not view the responses provided; and (4) respondent privacy would be strictly protected. The study questionnaires were stored securely, and data were entered into a password-protected database. Data were anonymized before the analysis by deleting all personal identifiers. The Faculty of Medicine of the Oita University Ethics Committee approved the study (approval no. 932).

### Statistical analysis

Students who did not complete the J-PSQI and the J-YDQ were excluded from the analysis. All analyses were stratified by gender. First, we plotted for the J-PSQI and J-YDQ score distributions. Second, participants were categorized as having a sleep disturbance of not according to their J-PSQI score, and categorized as not having IA, being at risk of IA, or having IA according to their J-YDQ Score. We calculated the prevalence of sleep disturbance according to IA status and determined whether there was a significant association between internet addiction and sleep disturbance using the chi-square test. Third, we conducted multiple logistic regression to measure the association between IA (as an explanatory variable) and sleep disturbance (as the dependent variable). The type of school, school-commute time, sports and club time, outside-class study time, television-viewing time, skipped meals, depressed mood, and school-life satisfaction were used as adjustment variables. The Statistical Package for Social Sciences Version 22 (SPSS, IBM Corp. NY, USA) for Windows was used for all statistical analyses. *P*-values < 0.05 were considered statistically significant.

## Results

**Fig. 1:**
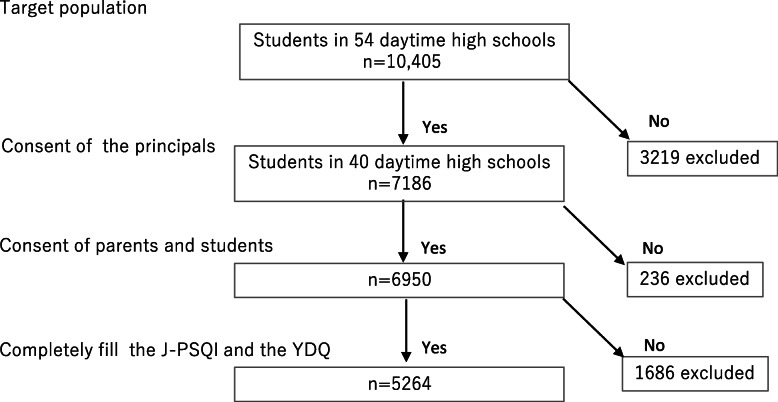
Flowchart of the participant selection process

Figure 1 shows a flowchart of the participant-selection process. Of the 54 schools (with a total of 10,405 students) that were requested to participate, 40 schools agreed to participate. At the time of the study, there were 7186 first-year (of the 3-year program) high school students, of whom 6950 provided informed consent or their parents provided consent (response rate: 96.7%). Of these, 5264 students (2635 boys and 2629 girls) completed the J-PSQI and-J-YDQ (effective response rate: 73.3%).

**Fig. 2:**
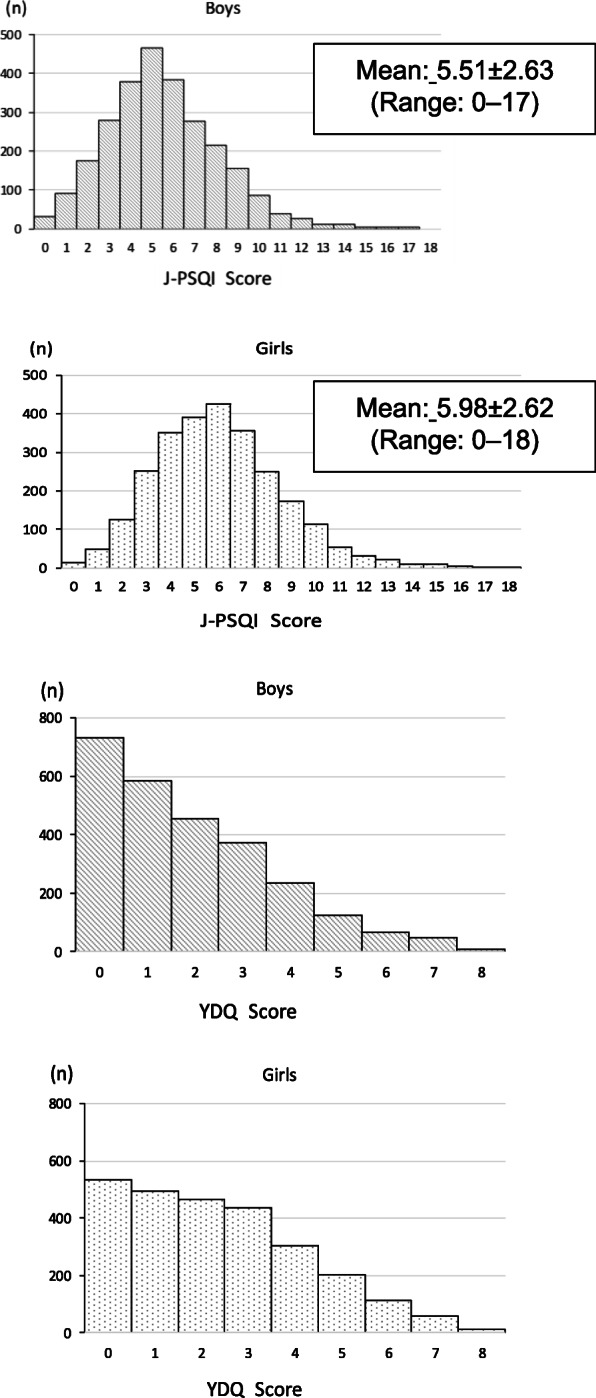
Distribution of the J-PSQI and YDQ Internet addiction scores. J-PSQI, Japanese version of the Pittsburgh Sleep Quality Index; YDQ, Young Diagnostic Questionnaire

Figure 2 shows the distribution of J-PSQI and YDQ scores. The J-PSQI scores for boys and girls were symmetrically distributed around a cutoff point value of 5.5 points. The mean and SD of the total J-PSQI score was 5.51 ± 2.63 (range: 0–17) and 5.98 ± 2.62 points (range: 0–18) for boys and girls, respectively. Regarding the YDQ scores, 0 was the most frequent score for both boys and girls. However, the point distribution varied by gender. Among boys, the number of points decreased as the score increased. Conversely, among girls, the score remained constant among those with 0 to 3 points and then gradually decreased as the number of points increased.

Table 1 shows the prevalence of students with sleep disturbance and the number of participants included in each of the three YDQ categories. Students defined as having sleep disturbance comprised 50.5% of all participants. In boys and girls, we observed a higher percentage of sleep disturbance in the following student groups: private high school students (*p* < 0.05), and those with a long school-commute time (*p* < 0.01), a high frequency of skipped meals per week (*p* < 0.001), depressed mood (*p* < 0.001), or poor school-life satisfaction (*p* < 0.001). Furthermore, boys spent little time engaging in school sports (or club) activities (*p* < 0.001). Regarding IA, the proportion with a YDQ score ≥ 5 and YDQ score of 3–4 was higher in girls than boys. In both boys and girls, the prevalence of IA was high in those with less than one-hour of school sport or club activity, more than seven skipped meals a week (*p* < 0.001), depressed mood (*p* < 0.001), and poor school-life satisfaction (*p* < 0.001). Additionally, the prevalence of IA was higher among girls who had shorter extracurricular learning time (*p* < 0.001). The relationship between television-viewing time and IA differed according to gender. The percentage of boys with a YDQ score ≥ 5 was higher among those who watched TV for ≥3 h per day. Conversely, the percentage of girls with a YDQ score ≥ 5 was higher among those who watched TV for ≤1 h per day.

**Table 1:** Percentage of sleep disturbance and the percentage of three YDQ categories

	n	Sleep disturbance		YDQ score			
		Yes	*p*-value	≤2	3–4	≥5	*p*-value
Total	5264	50.5%		62.3%	25.6%	12.1%	
Boys	2635	46.0%		67.4%	23.1%	9.4%	
**School type**			.047				.012
Public school	2017	45.0%		67.0%	24.2%	8.8%	
Private school	618	49.5%		68.8%	19.6%	11.7%	
**School-commute time (hours/day)**			.001				.962
< 0.5	1432	43.1%		67.9%	22.6%	9.6%	
≥ 0.5, < 1	947	48.4%		66.8%	23.8%	9.4%	
≥ 1	249	54.2%		66.7%	24.1%	9.2%	
**School sports or clubs (hours/day)**			<.001				<.001
None	757	52.2%		60.2%	26.8%	12.9%	
< 1	60	53.3%		50.0%	28.3%	21.7%	
≥ 1, < 2	204	47.1%		59.8%	26.0%	14.2%	
≥ 2	1601	42.7%		72.3%	20.9%	6.8%	
**Extracurricular learning (hours/day)**			.358				.072
None	454	44.5%		72.7%	17.6%	9.7%	
< 1	842	45.5%		66.3%	24.8%	8.9%	
≥ 1, < 2	864	45.4%		65.2%	24.8%	10.1%	
≥ 2	473	49.7%		68.5%	22.4%	9.1%	
**Television viewing time (hours/day)**			.156				.687
None	243	51.4%		66.7%	23.9%	9.5%	
< 1	485	46.2%		67.6%	23.3%	9.1%	
≥ 1, < 2	1049	44.2%		69.2%	22.3%	8.5%	
≥ 2, < 3	398	43.2%		68.1%	22.6%	9.3%	
≥ 3, < 5	324	50.3%		63.0%	24.7%	12.3%	
≥ 5	122	47.5%		63.1%	25.4%	11.5%	
**Skipped meals (times/week)**			<.001				<.001
None	1963	41.5%		69.7%	22.5%	7.8%	
< 2	335	57.6%		62.4%	24.5%	13.1%	
≥ 2, < 7	139	56.1%		61.2%	27.3%	11.5%	
≥ 7, < 14	78	66.7%		57.7%	20.5%	21.8%	
≥ 14	112	64.3%		58.0%	25.9%	16.1%	
**Depressed mood**			<.001				<.001
Not at all	555	29.0%		82.0%	14.8%	3.2%	
Not so much	906	39.3%		71.6%	23.5%	4.9%	
Yes	830	60.0%		59.6%	24.8%	15.5%	
Often	340	57.9%		51.2%	31.8%	17.1%	
**School-life satisfaction**			<.001				<.001
Satisfied	993	34.1%		77.2%	18.8%	3.9%	
More or less satisfied	1035	47.4%		67.0%	24.8%	8.2%	
Not really satisfied	413	62.5%		52.5%	27.1%	20.3%	
Dissatisfied	186	64.5%		50.5%	27.4%	22.0%	
Girls	2629	55.0%		57.1%	28.1%	14.8%	
**School type**			.034				.975
Public school	1980	53.8%		57.0%	28.2%	14.8%	
Private school	649	58.6%		57.5%	27.9%	14.6%	
**School-commute time (hours/day)**			<.001				.369
< 0.5	1212	52.4%		55.4%	28.8%	15.8%	
≥ 0.5, < 1	1092	55.0%		59.1%	26.8%	14.1%	
≥ 1	320	65.0%		56.6%	30.0%	13.4%	
**School sports or clubs (hours/day)**			.087				.373
None	1520	56.3%		55.2%	29.3%	15.5%	
< 1	70	61.4%		54.3%	28.6%	17.1%	
≥ 1, < 2	180	57.8%		60.6%	25.6%	13.9%	
≥ 2	842	51.7%		60.0%	26.6%	13.4%	
**Extracurricular learning (hours/day)**			.102				.001
None	259	61.8%		50.2%	30.1%	19.7%	
< 1	694	55.3%		53.0%	29.8%	17.1%	
≥ 1, < 2	954	53.1%		58.1%	28.7%	13.2%	
≥ 2	722	54.6%		62.2%	24.9%	12.9%	
**Television viewing time (hours/day)**			.071				.015
None	112	66.1%		51.8%	25.9%	22.3%	
< 1	362	56.4%		58.8%	25.4%	15.7%	
≥ 1, < 2	990	53.0%		59.4%	27.2%	13.4%	
≥ 2, < 3	515	53.2%		58.4%	29.7%	11.8%	
≥ 3, < 5	477	54.7%		54.5%	28.1%	17.4%	
≥ 5	158	60.8%		46.8%	34.8%	18.4%	
**Skipped meals (times/week)**			<.001				<.001
None	1688	48.6%		61.4%	25.8%	12.8%	
< 2	538	62.6%		51.5%	33.1%	15.4%	
≥ 2, < 7	203	72.9%		48.8%	32.0%	19.2%	
≥ 7, < 14	100	75.0%		46.0%	29.0%	25.0%	
≥ 14	98	64.3%		41.8%	32.7%	25.5%	
**Depressed mood**			<.001				<.001
Not at all	310	29.4%		78.4%	18.4%	3.2%	
Not so much	814	46.1%		65.1%	26.2%	8.7%	
Yes	1098	64.3%		48.1%	31.9%	20.0%	
Often	406	67.0%		49.0%	29.3%	21.7%	
**School-life satisfaction**			<.001				<.001
Satisfied	948	44.2%		67.0%	24.5%	8.5%	
More or less satisfied	1163	57.5%		55.1%	30.7%	14.2%	
Not really satisfied	371	68.5%		43.7%	30.5%	25.9%	
Dissatisfied	141	71.6%		41.8%	24.8%	33.3%	

Participants with missing data were excluded from the analysis. *P*-values were calculated using the χ^2^ test

Table 2 shows the prevalence of students with sleep disturbance for each of the three categories of YDQ. In both boys and girls, students categorized as having IA, or at risk of developing IA, had a higher prevalence of sleep disturbance.

**Table 2:** Prevalence of sleep disturbance according to gender and Young Diagnostic Questionnaire score

Gender	YDQ score	n	Sleep disturbance	
			%	95% CI
Boys				
	≤ 2	1776	38.6	36.3–40.9
	3–4	610	57.7	53.8–61.6
	≥ 5	249	70.3	64.6–76.0
Girls				
	≤ 2	1501	44.5	42.0–47.0
	3–4	739	63.9	60.4–67.4
	≥ 5	389	78.4	74.3–82.5

Abbreviations: *CI* confidence interval, *YDQ* Young Diagnostic Questionnaire

The YDQ scores are interpreted as follows: ≤ 2, no Internet addiction; 3–4, at risk of Internet addiction; ≥ 5, Internet addiction

Table 3 shows the multiple logistic regression analysis of the relationship between YDQ categories and sleep disturbance. In both boys and girls, the there was a statistically significant association between sleep disturbance and IA (Table 3). As compared to boys with YDQ scores ≤2, boys with YDQ scores of 3–4 and those with YDQ scores ≥5 had an increased likelihood of sleep disturbance (OR: 2.17, 95% CI: 1.80–2.61; and OR: 3.76, 95% CI: 2.82–5.01, respectively). Similarly, as compared to girls with YDQ scores ≤2, girls with YDQ scores 3–4 and those with YDQ scores ≥5 had an increased likelihood of sleep disturbance (OR: 2.20, 95% CI 1.84–2.64; and OR: 4.53, 95% CI: 3.48–5.88, respectively) The association between the YDQ scores and sleep disturbance remained significant (*p* < 0.001) after adjustment for potential confounding variables.

**Table 3:** Results of the multiple logistic regression analysis of the relationship between YDQ score and sleep disturbance

Gender	YDQ score	Sleep disturbance					
		OR	95% CI	*p*-value	AOR	95% CI	*p*-value
Boys							
	≤ 2	1.00			1.00		
	3–4	2.17	1.80–2.61	< 0.001	1.81	1.48–2.20	< 0.001
	≥ 5	3.76	2.82–5.01	< 0.001	2.37	1.74–3.23	< 0.001
Girls							
	≤ 2	1.00			1.00		
	3–4	2.20	1.84–2.64	< 0.001	1.93	1.59–2.33	< 0.001
	≥ 5	4.53	3.48–5.88	< 0.001	3.36	2.55–4.43	< 0.001

Abbreviations: *AOR* adjusted odds ratio, *CI* confidence interval, *OR* odds ratio, *YDQ* Young Diagnostic Questionnaire

The YDQ scores are interpreted as follows: ≤ 2, no Internet addiction; 3–4, at risk of Internet addiction; ≥ 5, Internet addiction

The AOR is adjusted for school type, school-commute time, school sports/clubs, extracurricular learning, television viewing time, skipped meals, depressed mood, and school-life satisfaction

## Discussion

This study aimed to clarify the relationship between sleep disturbance in adolescents and IA in one prefecture in Japan. We found an association between adolescent sleep disturbance and IA with sleep disturbance being more prevalent in boys and girls with higher YDQ scores. The results of the multivariate analysis revealed a significantly higher odds of sleep disturbance in students with high YDQ scores.

Despite the importance of adolescent sleep, sleep disturbance was present in more than half of the study participants. The high proportion of adolescents with sleep disturbance is in accordance with the results of recent studies of older adolescents using the PSQI [47, 48]. In this study, sleep disturbance was more frequent among students who did not participate in school sports or clubs, skipped meals, had depressed moods, and were dissatisfied with school-life. These results were similar to those of previous studies linking regular sleep habits to psychological and physical health [14]. Additionally, the prevalence of sleep disturbance was higher among students attending private schools and students with longer commutes. In general, students attending private schools tend to have longer commutes; therefore, they may have less sleep as has been shown previously [49]. Previous studies have suggested that sleep quality is related to health and emotions among youth [14, 50]. Future longitudinal studies should examine secular changes in these and other variables in order to improve the understanding of the relationship between sleep disturbance and Internet dependence.

Boys and girls who performed less than one school sport or club activity comprised the highest proportion of students with J-YDQ scores ≥5, providing evidence of a relationship between inactivity and IA. Additionally, students who skipped more than seven meals per week had a significantly higher likelihood of IA. Similar to previous studies [23, 26], the association of IA with exercise and diet was associated with a lack of a daytime routine. The proportion of high YDQ scores for extracurricular learning and TV viewing time differed between girls and boys. This may be attributable to gender-related differences in lifestyle and IA onset. In the multiple logistic regression analysis of the relationship between J-YDQ categories and sleep disturbance, the OR decreased after adjusting for lifestyle factors. Sleep disturbance and IA are associated with various lifestyle factors [23, 26]; therefore, this relationship needs further investigation. Future longitudinal surveys can be used to facilitate the development of health education programs to reduce the prevalence of sleep disturbance and Internet dependence.

The association between the YDQ scores and sleep disturbance is similar to that reported by Bakken et al. [51] from a study among Norwegians, aged ≥16 years in which participants with high YDQ scores had a significantly higher prevalence of sleep disturbance than non-problematic Internet users. Furthermore, this study found differences according to gender, with a stronger association between sleep disturbance and girls than in boys. This finding is similar to the results of a study by Durkee et al. [25], which found a significant relationship between insufficient sleep and IA in girls.

There are several possible mechanisms for the relationship between sleep disturbance and IA. First, a study by Tan et al. [22] found that IA could cause sleep disturbances. Moreover, Chen et al. [30] indicated that IA was associated with a disturbed circadian rhythm, leading to sleep disturbance.

Conversely, a second possible mechanism is that sleep disturbance might lead to the development of IA. In a longitudinal study, Chen et al. reported that falling asleep and nocturnal awakening difficulties were predictors of IA [30].

A third possible mechanism is that both conditions contribute to each other. Several studies on adults using brain imaging have confirmed that sleep disturbance and IA cause changes in the gray matter [52, 53]. A study of retired military personnel showed that individuals with a high PSQI score presented with a reduced volume of the entire cortex and frontal lobes, regardless of their mental health [53]. Another study that did not control for sleep disturbance reported that individuals with IA had reduced gray matter density [52]. These findings suggest that IA may cause organic (structural) changes in sleep-related neural pathways.

This study has the following three strengths: First, the sample size was adequate to ensure statistical power. Second, to investigate the relationship between sleep disturbance and IA, we used the PSQI and YDQ, which have been frequently used as standard indices in several epidemiological surveys [5, 10, 25, 39, 41, 43, 51, 52, 54–59]. Third, in our analysis, we evaluated the relationships between sleep disturbance and IA for each of the three categories of the YDQ, including at-risk Internet use.

This study also had several limitations. First, study was a cross-sectional survey, so it is not possible to formulate any conclusion regarding the direction of causality. Second, the analysis did not take into account schools as cluster units. Third, we did not adjust ORs for all the items that may be related to IA. For example, we did not ask questions regarding other psychiatric disorders, such as attention deficit hyperactivity disorder (ADHD), which has been reported to be associated with IA [60–62] and sleep disturbance [62] in adolescents. In this study, all participants attended daytime high school daily. In this setting, the number of students with ADHD is likely to be low. Fourth, students who were absent from school on the day of the survey could not participate. Fifth, our survey population was limited to students in a single prefecture in Japan; thus, it was a geographically limited population, so the results may lack generalizability. Finally, we did not investigate specific Internet-use disorders [4, 63–69], which need to be studied in detail for preventive measures to be developed.

## Conclusions

In summary, we observed that high Internet dependence was related to sleep disturbance in high school students in a prefecture in Japan. Studies have suggested that sleep disturbance and IA affect the gray matter in the brain. Longitudinal studies are required to further investigate the causal factors for IA and sleep disturbance and to clarify the mechanisms of their interdependence.

## Supplementary information

**Additional file 1.** Questions regarding daily-life habits. We present questions and responses from the survey form.

## Data Availability

The datasets generated and analyzed during the current study are not publicly available due to the sensitive nature of the raw data; however, all relevant study datasets are available from the corresponding author on reasonable request.

## Abbreviations

IA: Internet addiction
YDQ: Young Diagnostic Questionnaire
J-PSQI: The Japanese version of the Pittsburgh Sleep Quality Index
J-YDQ: The Japanese version of the Young Diagnostic Questionnaire

## References

[1] ITU (2017). ICT facts and figures.

[2] Mihara S, Osaki Y, Nakayama H, Sakuma H, Ikeda M, Itani O (2016). Internet use and problematic internet use among adolescents in Japan: a nationwide representative survey. Addict Behav Rep.

[3] Young KS (1998). Internet addiction: the emergence of a new clinical disorder. CyberPsychol Behav.

[4] Davis RA (2001). A cognitive-behavioral model of pathological internet use. Comput Hum Behav.

[5] Cheng C, Li AY (2014). Internet addiction prevalence and quality of (real) life: a meta-analysis of 31 nations across seven world regions. Cyberpsychol Behav Soc Netw.

[6] Dong G, Lu Q, Zhou H, Zhao X (2011). Precursor or sequela: pathological disorders in people with internet addiction disorder. PLoS One.

[7] Gamez-Guadix M, Calvete E, Orue I, Las HC (2015). Problematic internet use and problematic alcohol use from the cognitive-behavioral model: a longitudinal study among adolescents. Addict Behav.

[8] Lee BH, Lee HK (2017). Longitudinal study shows that addictive internet use during adolescence was associated with heavy drinking and smoking cigarettes in early adulthood. Acta Paediatr.

[9] Park B, Han DH, Roh S (2017). Neurobiological findings related to internet use disorders. Psychiatry Clin Neurosci.

[10] Morioka H, Itani O, Osaki Y, Higuchi S, Jike M, Kaneita Y (2017). The association between alcohol use and problematic internet use: a large-scale nationwide cross-sectional study of adolescents in Japan. J Epidemiol.

[11] Cao F, Su L (2007). Internet addiction among Chinese adolescents: prevalence and psychological features. Child..

[12] Yang SY, Lin CY, Huang YC, Chang JH (2018). Gender differences in the association of smartphone use with the vitality and mental health of adolescent students. J Am Coll Heal.

[13] Yang SY, Chen MD, Huang YC, Lin CY, Chang JH (2017). Association between smartphone use and musculoskeletal discomfort in adolescent students. J Community Health.

[14] Paruthi S, Brooks LJ, D'Ambrosio C, Hall WA, Kotagal S, Lloyd RM (2016). Recommended amount of sleep for pediatric populations: a consensus statement of the American Academy of sleep medicine. J Clin Sleep Med.

[15] Beebe DW, Rose D, Amin R (2010). Attention, learning, and arousal of experimentally sleep-restricted adolescents in a simulated classroom. J Adolesc Health.

[16] Brand S, Kirov R (2011). Sleep and its importance in adolescence and in common adolescent physical and psychiatric conditions. Int J Gen Med.

[17] Carskadon MA, Acebo C, Jenni OG (2004). Regulation of adolescent sleep: implications for behavior. Ann N Y Acad Sci.

[18] Casement MD, Keenan KE, Hipwell AE, Guyer AE, Forbes EE (2016). Neural reward processing mediates the relationship between insomnia symptoms and depression in adolescence. Sleep..

[19] Dahl RE, Lewin DS (2002). Pathways to adolescent health sleep regulation and behavior. J Adolesc Health.

[20] Fallone G, Owens JA, Deane J (2002). Sleepiness in children and adolescents: clinical implications. Sleep Med Rev.

[21] Wolfson AR, Carskadon MA (1998). Sleep schedules and daytime functioning in adolescents. Child Dev.

[22] Tan Y, Chen Y, Lu Y, Li L (2016). Exploring associations between problematic internet use, depressive symptoms and sleep disturbance among southern Chinese adolescents. Int J Environ Res Public Health.

[23] Alimoradi Z, Lin C-Y, Broström A, Bülow PH, Bajalan Z, Griffiths MD (2019). Internet addiction and sleep problems: a systematic review and meta-analysis. Sleep Med Rev.

[24] Yen CF, Ko CH, Yen JY, Cheng CP (2008). The multidimensional correlates associated with short nocturnal sleep duration and subjective insomnia among Taiwanese adolescents. Sleep..

[25] Durkee T, Carli V, Floderus B, Wasserman C, Sarchiapone M, Apter A (2016). Pathological internet use and risk-behaviors among European adolescents. Int J Environ Res Public Health.

[26] Yang SY, Chen KL, Lin PH, Wang PY (2019). Relationships among health-related behaviors, smartphone dependence, and sleep duration in female junior college students. Soc Health Behav.

[27] Wang PY, Chen KL, Yang SY, Lin PH (2019). Relationship of sleep quality, smartphone dependence, and health-related behaviors in female junior college students. PLoS One.

[28] Bartel KA, Gradisar M, Williamson P (2015). Protective and risk factors for adolescent sleep: a meta-analytic review. Sleep Med Rev.

[29] Kaneita Y, Ohida T, Osaki Y, Tanihata T, Minowa M, Suzuki K (2006). Insomnia among Japanese adolescents: a nationwide representative survey. Sleep..

[30] Chen YL, Gau SS (2016). Sleep problems and internet addiction among children and adolescents: a longitudinal study. J Sleep Res.

[31] Munezawa T, Kaneita Y, Osaki Y, Kanda H, Minowa M, Suzuki K (2011). The association between use of mobile phones after lights out and sleep disturbances among Japanese adolescents: a nationwide cross-sectional survey. Sleep..

[32] Itani O, Kaneita Y, Ikeda M, Kondo S, Yamamoto R, Osaki Y (2013). Disorders of arousal and sleep-related bruxism among Japanese adolescents: a nationwide representative survey. Sleep Med.

[33] Itani O, Kaneita Y, Munezawa T, Ikeda M, Osaki Y, Higuchi S (2016). Anger and impulsivity among Japanese adolescents: a nationwide representative survey. J Clin Psychol.

[34] Office of the Director General for Policy Planning for Policies on Cohesive Society CO (2013). International survey of youth attitude.

[35] Buysse DJ, Reynolds CF, Monk TH, Berman SR, Kupfer DJ (1989). The Pittsburgh sleep quality index: a new instrument for psychiatric practice and research. Psychiatry Res.

[36] Doi Y, Minowa M, Okawa M, Uchiyama M (1998). Development of the Japanese version of the Pittsburgh sleep quality index. Jap J Psych Treat.

[37] Doi Y, Minowa M, Uchiyama M, Okawa M, Kim K, Shibui K (2000). Psychometric assessment of subjective sleep quality using the Japanese version of the Pittsburgh sleep quality index (PSQI-J) in psychiatric disordered and control subjects. Psychiatry Res.

[38] American Psychiatric Association (1994). Diagnostic and statistical manual of mental disorders.

[39] Siomos KE, Dafouli ED, Braimiotis DA, Mouzas OD, Angelopoulos NV (2008). Internet addiction among Greek adolescent students. CyberPsychol Behav.

[40] Shek DT, Tang VM, Lo CY (2008). Internet addiction in Chinese adolescents in Hong Kong: assessment, profiles, and psychosocial correlates. Sci World J.

[41] Osada H (2013). Internet addiction in Japanese college students: is Japanese version of internet addiction test (JIAT) useful as a screening tool?. Bull Senshu Univ Sch Hum Sci Psychol.

[42] Laconi S, Rodgers RF, Chabrol H (2014). The measurement of internet addiction: a critical review of existing scales and their psychometric properties. Comput Hum Behav.

[43] Johansson A, Gotestam KG (2004). Internet addiction: characteristics of a questionnaire and prevalence in Norwegian youth (12-18 years). Scand J Psychol.

[44] Aboujaoude E (2010). Problematic internet use: an overview. World Psychiatry.

[45] Christos CF, Constantinos CF, Apostolos PK (2010). Internet addiction among Greek university students: demographic associations with the phenomenon, using the Greek version of Young's internet addiction test. Int J Econ Sci App Res.

[46] Dowling NA, Quirk KL (2009). Screening for internet dependence: do the proposed diagnostic criteria differentiate normal from dependent internet use?. CyberPsychol Behav.

[47] Alotaibi AD, Alosaimi FM, Alajlan AA, Bin Abdulrahman KA (2020). The relationship between sleep quality, stress, and academic performance among medical students. J Fam Community Med.

[48] Becker SP, Jarrett MA, Luebbe AM, Garner AA, Burns GL, Kofler MJ (2018). Sleep in a large, multi-university sample of college students: sleep problem prevalence, sex differences, and mental health correlates. Sleep Health.

[49] Hall WA, Nethery E (2019). What does sleep hygiene have to offer children's sleep problems?. Paediatr Respir Rev.

[50] Hosker DK, Elkins RM, Potter MP (2019). Promoting mental health and wellness in youth through physical activity, nutrition, and sleep. Child Adolesc Psychiatr Clin N Am.

[51] Bakken IJ, Wenzel HG, Gotestam KG, Johansson A, Oren A (2009). Internet addiction among Norwegian adults: a stratified probability sample study. Scand J Psychol.

[52] Zhou Y, Lin FC, Du YS, Qin LD, Zhao ZM, Xu JR (2011). Gray matter abnormalities in internet addiction: a voxel-based morphometry study. Eur J Radiol.

[53] Chao LL, Mohlenhoff BS, Weiner MW, Neylan TC (2014). Associations between subjective sleep quality and brain volume in gulf war veterans. Sleep..

[54] Lund HG, Reider BD, Whiting AB, Prichard JR (2010). Sleep patterns and predictors of disturbed sleep in a large population of college students. J Adolesc Health.

[55] Cheung LM, Wong WS (2011). The effects of insomnia and internet addiction on depression in Hong Kong Chinese adolescents: an exploratory cross-sectional analysis. J Sleep Res.

[56] Smagula SF, Stone KL, Fabio A, Cauley JA (2016). Risk factors for sleep disturbances in older adults: evidence from prospective studies. Sleep Med Rev.

[57] Kuss DJ, Griffiths MD, Karila L, Billieux J (2014). Internet addiction: a systematic review of epidemiological research for the last decade. Curr Pharm Des.

[58] Durkee T, Kaess M, Carli V, Parzer P, Wasserman C, Floderus B (2012). Prevalence of pathological internet use among adolescents in Europe: demographic and social factors. Addiction..

[59] Shek DT, Yu L (2012). Internet addiction phenomenon in early adolescents in Hong Kong. Sci World J.

[60] Yen JY, Ko CH, Yen CF, Wu HY, Yang MJ (2007). The comorbid psychiatric symptoms of internet addiction: attention deficit and hyperactivity disorder (ADHD), depression, social phobia, and hostility. J Adolesc Health.

[61] Ko CH, Yen JY, Chen CS, Yeh YC, Yen CF (2009). Predictive values of psychiatric symptoms for internet addiction in adolescents: a 2-year prospective study. Arch Pediatr Adolesc Med.

[62] Weinstein A, Yaacov Y, Manning M, Danon P, Weizman A (2015). Internet addiction and attention deficit hyperactivity disorder among schoolchildren. Isr Med Assoc J.

[63] Brand M, Young KS, Laier C, Wolfling K, Potenza MN (2016). Integrating psychological and neurobiological considerations regarding the development and maintenance of specific internet-use disorders: an interaction of person-affect-cognition-execution (I-PACE) model. Neurosci Biobehav Rev.

[64] Wu TY, Lin CY, Arestedt K, Griffiths MD, Brostrom A, Pakpour AH (2017). Psychometric validation of the Persian nine-item internet gaming disorder scale - short form: does gender and hours spent online gaming affect the interpretations of item descriptions?. J Behav Addict.

[65] Lin CY, Brostrom A, Nilsen P, Griffiths MD, Pakpour AH (2017). Psychometric validation of the Persian Bergen social media addiction scale using classic test theory and Rasch models. J Behav Addict.

[66] Lin CY, Imani V, Brostrom A, Arestedt K, Pakpour AH, Griffiths MD (2019). Evaluating the psychometric properties of the 7-item Persian game addiction scale for Iranian adolescents. Front Psychol.

[67] Lin C-Y, Imani V, Broström A, Nilsen P, Fung XCC, Griffiths MD (2019). Smartphone application-based addiction among Iranian adolescents: a psychometric study. Int J Ment Health Ad.

[68] Yam CW, Pakpour AH, Griffiths MD, Yau WY, Lo CM, Ng JMT (2019). Psychometric testing of three Chinese online-related addictive behavior instruments among Hong Kong university students. Psych Q.

[69] Lin C-Y, Lin C-K, Imani V, Griffiths MD, Pakpour AH (2020). Evaluation of the Selfitis behavior scale across two Persian-speaking countries, Iran and Afghanistan: advanced psychometric testing in a large-scale sample. Int J Ment Heal Addict.

