# Supplementary material for: Relationship between internet addiction and sleep disturbance in high school students: a cross-sectional study
Source: BMC Pediatr. 2020 Aug 11;20:379. doi: 10.1186/s12887-020-02275-7 (PMC7418409; doi:10.1186/s12887-020-02275-7)
Supplement: Supplementary file 1 — Additional file 1. Questions regarding daily-life habits. We present questions and responses from the survey form. [file 12887_2020_2275_MOESM1_ESM.pdf]

Additional file 1: Questions regarding daily life habits

Question	Responses
**School-commute time**<br>How much time do you spend traveling to school?	<0.5 hours<br>≥0.5 hours, <1 hour<br>≥1 hour, <2 hours<br>≥2 hours
**School sports or clubs**<br>Over the past months, how much time did you spend on average per day engaging in school sports or club activities?	None<br><1 hour<br>≥1 hour, <2 hours<br>≥2 hours.
**Extracurricular learning**<br>Over the past months, how much time did you spend on average per day in studying outside of school classes?	None<br><1 hour<br>≥1 hour, <2 hours<br>≥2 hours
**Television viewing time**<br>Over the past months, how much time did you spend on average per day watching television programs (including recorded programs)?	None<br><1 hour<br>≥1 hour, <2 hours<br>≥2 hours, <3 hours<br>≥3 hours, <5 hours<br>≥5 hours
**Skipped meals**<br>Over the past 30 days, did you ever skip (not eat) a meal or meals.	No<br><2 times/week<br>≥2times/week, <7times/week<br>≥7times/week, <14times/week<br>≥14times/week
**Depressed mood**<br>Over the past 30 days, did you have feelings of heaviness or depression more than usual?	Not at all<br>Not so much<br>Yes<br>Often
**School-life satisfaction**<br>Are you satisfied with your school life? Or are you dissatisfied with it?”	Satisfied<br>More or less satisfied<br>Not really satisfied<br>Dissatisfied
